# Wheat blast: The last enemy of hunger fighters

**DOI:** 10.1590/1678-4685-GMB-2022-0002

**Published:** 2023-04-03

**Authors:** Valeria Oliveira Nizolli, Vívian Ebeling Viana, Camila Pegoraro, Luciano Carlos da Maia, Antonio Costa de Oliveira

**Affiliations:** 1 Universidade Federal de Pelotas, Faculdade de Agronomia Eliseu Maciel, Departamento de Fitotecnia, Centro de Genômica e Fitomelhoramento, Pelotas, RS, Brazil.; 2 Universidade Federal de Pelotas, Faculdade de Agronomia Eliseu Maciel, Departamento de Fitossanidade, Pelotas, RS, Brazil.; 3 Universidade Federal de Pelotas, Centro de Desenvolvimento Tecnológico, Pelotas, RS, Brazil.

**Keywords:** Biotic stress, biotechnology tools, *Magnaporthe oryzae* L, *Triticum aestivum* L

## Abstract

Effective strategies for disease control are crucial for sustaining world food production and ensuring food security for the population. Wheat blast, a disease caused by the pathogen *Magnaporthe oryzae* pathotype *Triticum*, has been a concern for cereal producers and researchers due to its aggressiveness and rapid expansion. To solve this problem, the development of resistant varieties with durable resistance is an effective, economical and sustainable way to control the disease. Conventional breeding can be aided by several molecular tools to facilitate the mining of many sources of resistance, such as *R* genes and QTLs. The identification of new sources of resistance, whether in the wheat crop or in other cereals are an opportunity for efficient wheat breeding through the application of different techniques. Since this disease is still poorly studied in wheat, knowledge of the rice *Magnaporthe* pathotype may be adapted to control wheat blast. Thus, genetic mapping, molecular markers, transgenic approaches, and genomic editing are valuable technologies to fight wheat blast. This review aimed to compile the biotechnological alternatives available to accelerate the development of improved cultivars for resistance to wheat blast.

## Introduction

Wheat (*Triticum aestivum* L.) has a prominent role in the global economy, being associated with food security. In Brazil, wheat is the main winter crop, reaching 7.7 million ton in 2.73 million ha, resulting in an average yield of 2,803 kg ha-1 (CONAB, 2021; USDA, 2021). Although Brazil ranks 15th among the world’s largest wheat producers, the volume of wheat grown in the country is not capable of meeting internal consumption needs (USDA, 2021). Despite the great potential, the national cereal production scenario is affected by climatic factors, pests and diseases, causing restrictions to productivity (Souza and Vieira Filho, 2021). Diseases and pests are one of the main limitations to wheat production, causing reductions of 21% per year (Savary et al., 2019). Wheat blast, caused by *Pyricularia oryzae*, syn. *Magnaporthe oryzae*, pathotype *Triticum* (*MoT*), ranks first among the top 10 fungal pathogens in wheat based on its scientific/economic importance (Dean et al., 2012). Wheat blast appeared in Brazil in 1985, in the state of Paraná, and spread to other Brazilian states and later to several wheat-growing regions in Latin America (Urashima et al., 1993; Goulart et al., 2007). In 2016, *MoT* was reported in Bangladesh and, since then, it has been causing risks for wheat production on the Asian continent (Callaway, 2016; Cruz and Valent, 2017). By 2017, the disease had spread to India, one of the world’s largest wheat producers (Bhattacharya and Mondal, 2017; Yesmin et al., 2020). In 2017-2018, *MoT* was identified in Zambia, on the African continent (Tembo et al., 2020).

To control blast, genetic resistance is considered the most efficient and sustainable way. However, genetic resistance is a challenge for breeders and plant pathologists, due to the high diversity of *MoT* and the complexity of resistance inheritance. In this scenario, it is essential that cereal breeders use genomic science tools to develop strategies to control the disease in an effective and lasting way.

For the identification of genes as sources of resistance, as well as closely linked molecular markers, the mapping of Quantitative Trait Loci (QTLs) presents itself as a powerful tool. Likewise, transcriptomic analysis as RNASeq, Microarrays and quantitative real-time PCR help to identify routes and genes associated with genetic resistance (Kumar et al., 2020). The marker assisted selection (MAS) is one of the most used tools of genomic science and its application is a strategy for improving germplasm and the development of new blast resistant cultivars (He et al., 2020). In recent years, gene editing has gained notoriety due to its ability to enable the development of new genotypes through the modification of genes and/or DNA segments, resulting in the modification of interest, such as the plant resistance response to *MoT* infection (Zhu et al., 2017).

Finally, transgenics have already proven effective in improving other traits and can be considered as an option also in the search for durable resistance (Cruz and Valent, 2017). Therefore, this work aims to compile the available alternatives of genomic science to accelerate the development of *MoT* resistant wheat cultivars.

## Wheat blast

**Figure 1 -:**
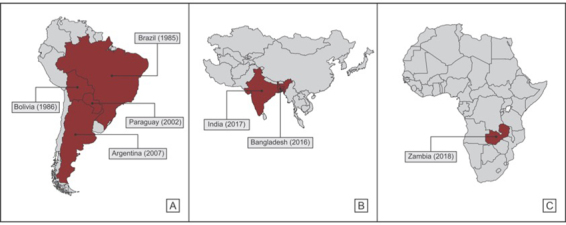
Blast occurrence in different countries. A. Countries in the South American affected by blast and the year of the disease arrival; B. Countries in Asia affected by blast and the year of the disease arrival; C. Country on the African continent affected by blast and the year of the disease arrival.

Wheat blast originated in Brazil and initially spread to Bolivia, Paraguay and Argentina (Urashima et al., 1993; Cruz and Valent, 2017; Ceresini et al., 2018; Singh et al., 2021). Currently, pathogen strains from South America are identified as causing the disease in countries on other continents (Singh *et al.*, 2021) (Figure 1). 

Originally the blast disease was identified in rice (*Oryza sativa* L.), caused by *Pyricularia oryzae*, syn. *Magnaporthe oryzae* pathotype *Oryzae* (*MoO*). *Magnaporthe oryzae* has an extensive host range and is responsible for causing blast disease in 50 species belonging to the Poaceae family, including the main cereal crops, such as wheat and barley (Gladieux et al., 2018; Langner et al., 2018; Valent *et al.*, 2019). Previous reports have shown that a single species of the pathogen is associated with the disease and is divided into strains/pathotype that are adapted to different hosts (Gladieux *et al.*, 2018; Valent *et al.*, 2019). In other words, individual pathogen isolates tend to be associated with a single Poaceae genus, showing specific genetic divergence among different host genotypes (Bentham et al., 2021). In wheat (*Triticum aestivum* L.), blast is caused by a strain of *M. oryzae Triticum* (*MoT*) that is adapted to cause disease in hosts of the *Triticum* genus, and this strain is not able to cause infection in rice crops (Maciel et al., 2014; Cruz and Valent, 2017). However, due to the wide range of hosts (multihosts), crossings between isolates from different strains can result in gene flow between them (Yesmin et al., 2020). 

Recently, Paul et al. (2022) demonstrated that the *MoO* pathotype, in a controlled environment, was able to infect wheat leaves and ears, whereas *MoT* was unable to infect rice plants. In this way, the study revealed the vulnerability of wheat, not only to *MoT*, but also to the *MoO* pathotype. As well as providing evidence of a potential *MoO* wheat blast epidemic in many rice and wheat growing regions with worsening climate changes. Thus, understanding of rice *Magnaporthe* pathotype may be adapted to control wheat blast.

The reproduction strategy of *MoT* is another feature that constrains blast control. Studies have reported occurrences of sexual reproduction of *MoT* in the field, which suggests that in wheat, unlike rice, the pathogen follows a mixed reproduction system (Maciel et al., 2014; Cruz and Valent, 2017; Yesmin et al., 2020).

Pathogens that have a mixed reproductive system, in which sexual recombination is followed by asexual recombination, are considered to have high evolutionary potential and, consequently, more difficult to control due to the dispersion of better adapted clones (Cruz and Valent, 2017; Yesmin et al., 2020).

**Figure 2 -:**
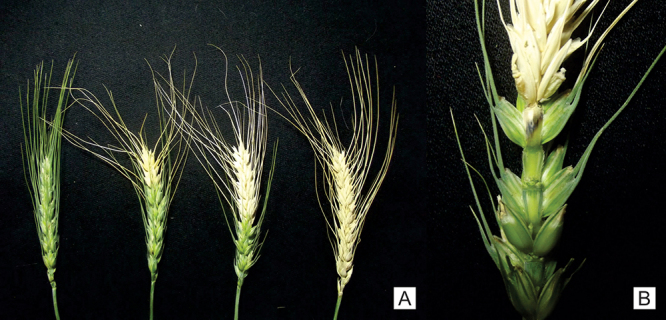
Blast symptom in wheat ears. A. Evolution of blast symptoms in wheat ears; B. Effect on wheat grain formation due infection by *Magnaporthe oryzae* pathotype *Triticum* in rachis.

Wheat blast is considered one of the main diseases that affect the wheat crop. *MoT* can cause reductions in grain yield and quality, leading to production losses of up to 100% (Cruz and Valent, 2017; Singh et al., 2021). The disease can occur in all parts of the plant, but in the ear its high incidence has the greatest impact (Cruz and Valent, 2017; Islam et al., 2020). The blast symptom found in ears is the total or partial bleaching of the ear above the point of infection (Figure 2), which occurs due to the colonization of the fungus in the tissue, preventing nutrient transport and impairing grain filling (Islam *et al.*, 2020; Singh *et al.*, 2021).

Taking into account all the characteristics of the disease mentioned above, genetic resistance is considered the best alternative for blast control. In the search for controlling strategies, plant genetic improvement plays an extremely important role due to the identification of genetic resistance sources and its use in the development of cultivars that are effectively resistant to the pathogen (Valent *et al.*, 2019; Islam et al., 2020). Plant defense reactions to attack by pathogens can be characterized by the number of genes that govern them, and may be specific against pathogen races (vertical) and non-specific (horizontal) (Savadi et al., 2018). In wheat, mainly the horizontal resistance has been detected, indicating moderate resistance responses with variation among different pathogen isolates and in different environments. This resistance response is governed by several minor effect genes (QTLs), acting together and is generally more difficult to overcome (Maciel et al., 2014). In this sense, the search for genes / QTLs of resistance (*R*) to *MoT* has been intense over the last decades.

## Sources of resistance

### Genes involved with wheat blast resistance

To date, a total of 10 *R* genes have been identified for wheat blast (Table 1) (*RmgTd(t)*, *Rmg1*(*Rwt4*), *Rmg2*, *Rmg3*, *Rmg4*, *Rmg5*, *Rmg6*(*Rwt3*), *Rmg7*, *Rmg8* and *RmgGR119*) (Takabayashi et al., 2002; Zhan et al., 2008; Nga et al., 2009; Vy et al., 2014; Anh et al., 2015; Tagle et al., 2015; Anh*et al.*, 2018; Wang S et al., 2018). However, these genes are specific against pathogen races and their expressions depend on the stage of the plant development and temperature, that should be high (~25 °C) to be ideal for infection and disease development (Cruz and Valent, 2017; Islam et al., 2020). The *Rmg2* and *Rmg3* genes are not effective in the head stage and at elevated temperatures. *Rmg7* and *Rmg8* provide resistance in the seedling and head stages and both are active at low temperatures (21-24 °C). However, at elevated temperatures (26 °C), *Rmg7* loses its role in resistance, while *Rmg8* remains effective (Tagle *et al.*, 2015; Anh *et al.*, 2018). *RmgGR119*, as well as *Rmg7* and *Rmg8*, confers resistance against the ear infection by *MoT,* and there are still no cases of overcoming this resistance (He et al., 2020). Therefore, the most promising genes for use in breeding programs for *MoT* resistance are *Rmg8* and *RmgGR119*, although *RmgGR119* has been shown to act additively with *Rmg8* for resistance against the Brazilian isolate BR48 of *MoT* (Wang S *et al.*, 2018; Inoue et al., 2021). The combination of these genes has the potential to confer a more durable and efficient resistance (Islam *et al.*, 2020). 

**Table 1 -:** Sources of resistance to wheat blast.

Gene name	Resistance source	Pathogen isolate	Reference
*RmgTd(t)*	*T. dicoccoides* KU109 (Tat4)	Hidden gene	Takabayashi et al. (2002)
*Rmg1(Rwt4)*	*T. aestivum*, Norin 4	Oat Isolate Br58	Takabayashi et al. (2002)
*Rmg2*	*T. aestivum*, Thatcher	Wheat isolate Br48	Zhan et al. (2008)
*Rmg3*	*T. aestivum*, Thatcher	Wheat isolate Br49	Zhan et al. (2008)
*Rmg4*	*T. aestivum*, Norin 4	Digitaria isolate	Nga et al. (2009)
*Rmg5*	*T. aestivum*, Red Egyptian	Digitaria isolate	Nga et al. (2009)
*Rmg6(Rwt3)*	*T. aestivum*, Norin 4	Ryegrass isolated TP2	Vy et al. (2014)
*Rmg7*	*T. dicoccum*, KU120; *T. dicoccum*, KU112; *T. dicoccum*, KU122	Wheat isolate Br48	Tagle et al. (2015)
*Rmg8*	*T. aestivum*, S-615	Wheat isolate Br49	Anh et al. (2015); Anh *et al.* (2018)
*RmgGR119*	Albanian Wheat Access GR119	Wheat isolate Br50	Wang S et al. (2018)
Translocation *2NS*	Chromosomal segment of *Aegilops ventricosa*	Wheat isolate Br48	Cruz et al. (2016)

Source: Adapted from Islam et al. (2020).

In addition to these genes, a 2NS chromosomal segment translocation was identified in *Aegilops ventricosa* as a source of resistance to wheat blast (Cruz et al., 2016) (Table 1). Its presence has been identified in genotypes with a higher level of resistance to *MoT,* and its efficacy has been confirmed in natural epidemic conditions (Cruz *et al.*, 2016; Cruppe et al., 2021; Islam et al., 2020). However, this resistance is not effective for some *MoT* isolates (Inoue et al., 2021).

Concerning the resistance sources to be used in genetic improvement, an important aspect is their durability. The durability of the resistance conferred by the *R* genes is evaluated based on their corresponding *AVR* genes, the effectors known as avirulence molecules (*AVRs*) that are present in the pathogen (Hafeez et al., 2021; Inoue et al., 2021). The *R* genes encode immune receptors that directly or indirectly recognize *AVRs*, and trigger the initiation of defense responses capable of limiting the pathogen proliferation in the host (Hafeez *et al.*, 2021). An example is the *AVR-Rmg8* gene, which was conserved in *MoT* isolates, that is recognized by the protein encoded by the *Rmg8* gene (Wang S et al., 2018). A recent study suggested that the effect of *Rmg8* is suppressed by the effect of the *PWT4* gene, the corresponding *AVR* gene of *Rmg1*(*Rwt4*), which was transferred horizontally from *P. pennisetigena* isolates to *P. oryzae* isolates from oat (Inoue *et al.*, 2021). The transfer of the *PWT4* to *MoT* isolates would imply a potential risk of overcoming the resistance conferred by *Rmg8*. This is because, *PWT4* suppresses *Rmg8*-mediated resistance in wheat or interferes with recognition of *AVR-Rmg8* by *Rmg8*. Thus, to avoid an irreparable loss of wheat resistance to *MoT*, it is recommended that *Rmg8* be introduced together with *Rmg1*(*Rwt4*) (Inoue *et al.*, 2021). 

The identification of other sources of resistance to *MoT* is of high importance due to the risk that the disease will spread to other wheat-producing regions, threatening world food security (Cruz and Valent, 2017; Islam et al., 2020). Therefore, it is necessary to use technologies such as QTL mapping and gene expression analysis. A larger number of genomic sequences and genes involved in resistance can help the plant genetic improvement process, enabling a variety of combinations in order to slow down the pathogen’s co-evolution and to improve the durability of resistance to wheat blast.

### Gene pool

The phenomenon of polyploidization that generated bread wheat led to a restricted genetic diversity (polyploidization bottleneck), making wheat breeding a challenging task, when compared to other crops (Ali et al., 2008; Venske et al., 2019). In addition to this, domestication, centuries of cultivation and the selection pressure exerted within crop breeding programs has favored a reduction in genetic diversity (Savadi et al., 2018; Venske *et al.*, 2019). Wheat breeding programs around the world rely on limited germplasm, resulting in wheat cultivars that comprise only 10% of existing wheat diversity (Savadi *et al.*, 2018).

Under these conditions, an important alternative to increase genetic diversity is the use of species from the secondary and tertiary gene pools as donors of new genes and alleles (Ceresini et al., 2018; Venske et al., 2019; Tosa, 2021). These are important sources of resistance genes to different biotic and abiotic stresses. However, the use of these sources of diversity is complicated due to problems related to crossover incompatibility, hybrid sterility, in addition to the linkage of several undesirable characters with desirable ones (linkage drag).

Despite having these disadvantages when one considers conventional (classic manual crossing), to explore the gene pools as resistance gene donors through genetic transformation and genome editing can be used (Venske et al., 2019; Sharma et al., 2021). Furthermore, another alternative to increase genetic diversity is mutation inducing, which enables the creation of new alleles.

Most of the resistance genes (and chromosomal segment) against wheat blast fungus come from related species, such as *Triticum dicoccoides* and *Aegilops ventricosa* (Table 1). To find new genes, *Triticum*-*Aegilops* accessions have been explored, such as *T. boeoticum*, *Ae. tauschii*, *Ae. umbellulata*, *Ae. comosa*, and *Ae. uniaristata*, even if still without promising results (Tosa, 2021).

In rice, a greater number of genes for resistance to blast have been documented, there are also examples of *R* genes originating from documented *Oryza* species. The genes *Pirf2-1(t)* from *O. rufipogon*, *Pi-40(t)* from *O. australiensis*, *Pi-13* from *O. minuta*, *Pid3-A4* from *O. rufipogon*, *Pi54rh* from *O. rhizomatis* and *Pi54of* from *O. officinalis* (Amante-Bordeos et al., 1992; Jeung et al., 2007; Utani et al., 2008; Das et al., 2012; Lv et al., 2013; Devanna et al., 2014). However, to better explore these sources of diversity, it is necessary that screening for blast resistance should be performed under controlled conditions, using a representative selection of virulent pathogen isolates (Ceresini et al., 2018).

Another alternative would be to explore familiar crops such as oats, rice and barley. Using bioinformatics, it is possible to find differentially expressed genes for the blast stress in these crops that are corresponding to wheat (Cruz and Valent, 2017; Tosa, 2021).

### Molecular markers and QTL mapping

The development and application of molecular markers in plant genetic improvement have helped breeders during the development process of new cultivars, enabling the selection of plants with desirable traits in a precise way, minimizing the effects of the environment (Devanna et al., 2014). In the main cultivated species, it is possible to find examples of the application of molecular markers related to resistance to pathogens (Islam et al., 2020). However, due to the wide variety of markers available, it is important to understand how this technology works to better apply it. From the creation of molecular markers, it was possible to apply them in QTL mapping, in marker assisted selection (MAS), and as an auxiliary tool in gene editing and genetic transformation, to produce superior plants. In addition, molecular markers can be used to assess genetic diversity, parental assessment, germplasm characterization, among other applications (Idrees and Irshad, 2014; Kordrostami and Rahimi, 2015; Islam *et al.*, 2020). 

In rice breeding for blast resistance, SNP markers were developed for the *R* genes *Piz*, *Piz-t*, *Pit*, *Pik*, *Pik-m*, *Pikp*, *Pita*, *Pita-2* and *Pib* (Hayashi et al., 2004). In wheat, SNPs have been used in several Genome-Wide Association Studies (GWAS) for blast resistance (Islam et al., 2020; Juliana et al. 2020; Cruppe et al., 2021; He et al., 2021a).

The mapping of economically important genes, based on genetic maps, has been supporting breeding programs for a wide range of plant species (Ashkani et al., 2016). Such importance is due to the fact that genetic mapping is able to locate and identify genes of a quantitative nature, which contributed to the variation of complex traits, such as disease resistance (Stich and Melchinger, 2010). For blast, more than 350 QTLs were mapped in rice (Ashkani *et al.*, 2016; Devanna et al., 2014). However, the number of studies on QTL mapping that confer resistance to wheat blast resistance is still low (Table 2), and few of those available have identified major and stable QTLs beyond the 2AS / 2NS translocation (Singh et al., 2021).

**Table 2 -:** QTL studies in mapping for wheat blast resistance.

Linkage mapping			
QTL number	DNA markers	Mapping population	Reference
*QWbr.emt-2ª*	KASP and SSRs	Backcross population	Ferreira et al., 2021
*QPag.emt-2ª*	KASP and SSRs	Backcross population	Ferreira et al., 2021
*QWbr.emt-5B*	KASP and SSRs	Backcross population	Ferreira et al., 2021
*QWbr.emt-7B*	KASP and SSRs	Backcross population	Ferreira et al., 2021
Loco 2AS	DArTSeq and STS	Backcross population	He et al., 2021b
Loco 2DL	DArTSeq and STS	Backcross population	He et al., 2021b
Loco 7AL	DArTSeq and STS	Backcross population	He et al., 2021b
Loco 7DS	DArTSeq and STS	Backcross population	He et al., 2021b
Association mapping			
QTLs number DNA markers Mapping population Reference			
Loco 2AS	SNP	Designed panel	Juliana et al., 2020
Loco 3BL	SNP	Designed panel	Juliana et al., 2020
Loco 4AL	SNP	Designed panel	Juliana et al., 2020
Loco 7BL	SNP	Designed panel	Juliana et al., 2020
Loco 1AS	STS	Designed panel	He et al., 2020
Loco 2BL	STS	Designed panel	He et al., 2020
Loco 3AL	STS	Designed panel	He et al., 2020
Loco 4BS	STS	Designed panel	He et al., 2020
Loco 4DL	STS	Designed panel	He et al., 2020
Loco 7BS	STS	Designed panel	He et al., 2020
Loco 2A	SNP	Designed panel	Cruppe et al., 2021
Loco 1BS	SNP and STS	Designed panel	He et al., 2021a
Loco 2AS	SNP and STS	Designed panel	He et al., 2021a
Loco 6BS	SNP and STS	Designed panel	He et al., 2021a
Loco 7BL	SNP and STS	Designed panel	He et al., 2021a
Loco 1A	SNP	Designed panel	Goddard et al., 2020
Loco 2B	SNP	Designed panel	Goddard et al., 2020
Loco 4A	SNP	Designed panel	Goddard et al., 2020
Loco 5A	SNP	Designed panel	Goddard et al., 2020

As several *R* genes have already been shown to be less effective against recent *MoT* isolates, combining sources of resistance will be essential to prevent further outbreaks of the disease. An example is a QTL unrelated to the 2AS / 2NS translocation involving BR 18-Terena, one of the Brazilian wheat genotypes with the highest level of resistance to *MoT* available in Brazil. BR 18-Terena has a quantitative resistance to wheat blast, and nine QTLs associated with resistance at the seedling and heading stages were detected (Table 2) (Goddard et al., 2020). QTLs are essential sources of resistance to retard the evolution of virulent *MoT* isolates. Thus, it is fundamental to amplify efforts for the identification of QTLs, especially those unrelated to the 2AS / 2NS translocation.

Recent studies that have been carried out in the field have shown that resistance to *MoT* is quantitative and that 2NS translocation explains much of the disease’s variation in different environments. A GWAS study was performed to identify genomic regions associated with resistance to MoT in the field (Juliana et al., 2020). In this study, 36 markers associated with blast resistance were identified on chromosomes 2AS, 3BL, 4AL and 7BL, with more than half of them marking the 2NS translocation and explaining up to 71.8% of the variation for the disease. As in the previous study, a report on the 2NS translocation explaining 22.4-50.1% of disease variation in diverse environments in their mapping population was described (He et al., 2020). A QTL on chromosome 2AS explaining an average of 84.0% of the phenotypic variation in response to *MoT* was reported, reinforcing the potency of the 2NS translocation (Ferreira et al., 2021). He *et al.* (2021a) found that the 2NS translocation was the only major and consistent resistance locus, while loci in other chromosomal regions had low phenotypic effects and were not stably expressed in the experiments. In these studies, to verify the presence of the 2NS / 2AS translocation, the recommended markers are Ventriup-LN2 reported by Helguera et al. (2003), WGGB156 and WGGB159 by Wang et al. (2018) and cslVrgal3, derived from a study by Seah et al. (2001). However, there are still few markers related to major and stable QTLs beyond the 2AS / 2NS translocation (He *et al.*, 2020; Juliana *et al.*, 2020; He*et al.*, 2021b; Singh et al., 2021).

## Breeding for blast resistance

### Conventional breeding and mutation breeding

The genetic improvement of plants is one of the tools used to change plant traits in order to improve their qualities for human benefit, such as increasing cultivar yields and maintaining food security (Stich and Melchinger, 2010). Pathogens such as *MoT* pose a current and growing threat to food security and, to mitigate the problem, breeding strategies aimed at creating disease resistant cultivars are essential. In conventional breeding, the presence of genetic variability in the parents to be recombined influences directly the success of the program. 

Therefore, sources of resistance genes, such as elite cultivars, germplasm collections, wild species and mutation induction must be explored. 

For wheat blast, the genetic basis of resistance is not well defined due to the wide variation in the virulence scale (Urashima et al., 2004). However, we can find some cultivars that show moderate levels of resistance against *MoT*, such as the Brazilian cultivars IPR 85, CD 113 and BR-18 Terena. Other cultivars can be found around the world, such as the Bolivian cultivar named Paragua CIAT and Parapeti CIAT, which showed a high level of resistance when compared to the others. However, cultivar Milan, from CIMMYT (International Center for Maize and Wheat Breeding) showed the highest level of resistance among all resistant cultivars described (Kohli et al., 2011; Islam et al., 2020). In Brazil, this year, the cultivar TBIO Triunfo (Biotrigo Genetica) was released, moderately resistant to blast, an example of the result of conventional genetic improvement work for the trait in the country. In Japan, breeding programs have been introducing blast resistance genes into their elite wheat varieties (Wang M et al., 2018). 

Conventional breeding, even with its indispensable techniques and a history of great achievements in plant science, faces several challenges regarding wheat blast. In the conventional model, the process is challenged by the time required (5-10 years), the availability of genetic resources resistant to *MoT*, and the difficulty in selecting for resistance of a quantitative nature (Savadi et al., 2018; Islam et al., 2020). Even so, with the help of biotechnology tools, such as molecular markers, this process can have better results, especially in backcrosses with gene pyramiding. Also, accelerated breeding schemes, such as speed breeding, can generate faster results (Watson et al., 2018).

In view of the limited sources of resistance available to *MoT* and the restricted genetic variability of wheat, inducing artificial mutations to create genetic variability is an alternative (Zhu et al., 2017). For this purpose, several mutation induction approaches can be used, with methods involving physical (X-ray, gamma radiation, ultraviolet radiation) and chemical (colchicine, EMS - ethylmethanesulfonate, MMS - methylmethanesulfonate (Hussain, 2015) mutagens. This is a difficult process, as the methods cause random mutations and provide a limiting mutation frequency at the desired/target loci, leading to uncertain results (Zhu *et al.*, 2017; Ijaz et al., 2020) be considered.

**Figure 3 -:**
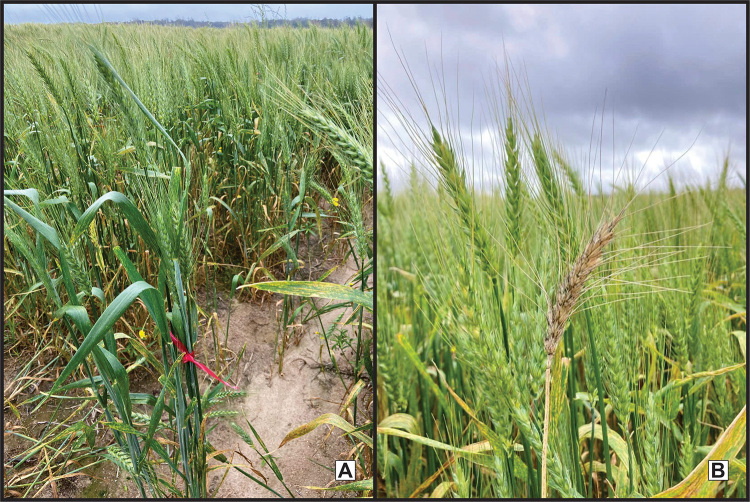
A. M_4_ mutant plant showing resistant phenotype; B. M_4_ individual showing susceptible phenotype. Credits to Amanda Valentini Baseggio.

The induction of mutations aimed at resistance to blast in wheat has been hardly explored, but some studies have been reported in the literature. Induced mutations in wheat lines using gamma rays to obtain *MoT* resistant genotypes was performed (Harun-Or-Rashid et al., 2019). Among the evaluated varieties, BARI Gom-30 showed the best performance in M_2_. However, the mutant lines need to be advanced a few generations, artificially inoculated with the *MoT* pathogen, and evaluated for resistance to confirm the results. The authors emphasize that this approach can be a potential substitute for the available chemical control methods, being described as an ecological and sustainable strategy. A study entitled Disease Resistance in Rice and Wheat for Better Adaptation to Climate Change, funded by the FAO, is currently being conducted. This research has the efforts of researchers from 10 countries, and one of the goals is the identification of *MoT*-resistant wheat mutants (IAEA, 2018). Our group initiated induced mutation for blast resistance in wheat in 2018, and the parental variety was TBIO Toruk. Several thousand plants have been conducted after 2 gamma-ray (250 and 300Gy) and one chemical (2% EMS) treatments. Some mutant individuals seemed resistant after inoculation with *MoT* race *Pyricularia oryzae* 4-06 (Figure 3).

In a recent study, alleles that may confer resistance to *MoT* in a mutant population of wheat in M_2_ were detected, providing valuable information for genetic improvement (Guo et al., 2021). The population, exposed to the EMS mutagen, was analysed by TILLING (Targeting Induced Local Lesions IN Genomes). The study revealed 81 SNPs located in exonic and promoter regions, as well as 13 alleles related to resistance to *MoT*.

### Molecular breeding

The integration of biotechnology strategies in the improvement of agricultural crops has contributed to the development of disease resistance, accelerating the development of new varieties. Among the biotechnology tools that have direct application and great impact on plant breeding, molecular markers and transgenics, and more recently the genome editing technique using CRISPR technology, can be highlighted.

*Transgenics*

In plant breeding, transgenesis has been considered a powerful tool, capable of introducing a wide range of desirable traits into agricultural crops, such as higher yield and nutrition, and tolerance to biotic and abiotic stresses (Periyannan et al., 2013; Chang et al., 2016; Savadi et al., 2017; 2018).

Using transgenics, breeders can precisely manipulate the gene that encodes a trait of interest, inserting genes from unrelated species or silencing specific genes (Miah et al., 2013). Research using transgenics has shown promissing results for genetic resistance to diseases caused by several pathogens, such as *Blumeria graminis*, *Bipolaris sorokiniana*, *Fusarium graminearum*, *Rhizoctonia solani* and *Pyricularia oryzae* (Shimono et al., 2007; Helliwell et al., 2013; Li et al., 2015; Del Ponte et al., 2017). For blast, several transgenic studies were performed aiming to develop plants resistant to the disease, most of them carried out in rice (Shimono *et al.*, 2007; Chen et al. 2010; Helliwell *et al.*, 2013; Bundó and Coca, 2016; Wang and Valent, 2017; Chandran et al., 2019).

In rice, overexpression of the transcription factor *WRKY45* was explored in relation to blast resistance by Shimono et al. (2007). In another study with transgenic rice, *ACS 2* overexpressing lines were more resistant to blast due to high ethylene production and expression of *PR1b* and *PR5* genes (Helliwell et al., 2013). In millet, genetic transformation of a rice *chitinase* gene (*chi11*) showed resistance to leaf blast (Ignacimuthu and Ceasar, 2012). Even with so many promising examples, there are still no applications of this technology to develop *MoT*-resistant transgenic wheat lines. 

There are still many controversies involving transgenic organisms, especially in crops, such as wheat, where the acceptance of the technology is still a matter of debate. An important point to consider in the development of transgenic plants is the production time and adaptation to the legislation that the product demands, from its discovery until it becomes a commercial product, which would take approximately 10 years of research. Even so, there are countless possibilities involving this technology, such as the development of transgenic plants overexpressing genes involved in the resistance response to high-impact diseases, such as wheat blast.

In Brazil, only six species with transgenic events are released for cultivation, namely: maize, soybean, cotton, bean, sugarcane and eucalyptus. In the world, until recently, only wheat with a transgenic event for tolerance to the herbicide Glyphosate had been released for cultivation in Australia, United States, New Zealand and Colombia (ISAAA, 2022). However, in October 2020, Argentina’s Ministry of Agriculture approved the GM *HB4* drought-tolerant wheat for cultivation and consumption, making it the first country to adopt *HB4* technology (Sheridan, 2021). This release had an impact on Brazil, Argentina’s main wheat importer, where, in recent days, the marketing of wheat flour produced from the genetically modified cereal was approved by the National Technical Biosafety Commission (CTNBio), the entity responsible for regulating GMOs in the country. Despite the controversy surrounding the decision, the release of transgenic wheat for cultivation and consumption is a great advance and reinforces its applicability of the technology for other diverse purposes in wheat cultivation.

*Gene editing*

Technologies aimed at changing the DNA are not new. Since the double helix structure discovery in 1953, technological advances have advanced rapidly (Watson and Crick, 1953). At first, DNA changes were performed by random mutagenesis using chemical or physical methods (Zhu et al., 2016; Kun et al., 2019), which, despite being random, are still frequently used today. In a second moment, technologies were developed that enabled the silencing or random insertion of genes into the genome (transgenesis), as mentioned above (Wang et al., 2017). Finally, it is possible to find highly accurate gene editing technologies (Jinek et al., 2012; Lino et al., 2018).

Editing strategies use sequence-specific nucleases (SSNs), which promote the generation of double-strand DNA breaks (DSBs) at specific locations within the genome in a mediated manner. DSB repair can be performed by two cellular repair mechanisms: homologous recombination - HR and non-homologous end joining - NHEJ (Zhu et al., 2017; Viana et al., 2019). Different editing tools have been used, such as Mega nucleases (MNs), Zinc finger nucleases (ZFNs), Transcription activator-like effector nucleases (TALENs) and Clustered regularly interspaced short palindromic repeat/CRISPR-associated protein (CRISPR / Cas9) (Zhu *et al.*, 2017; Viana *et al.*, 2019). MNs have been successfully used in some species, such as *Arabidopsis*, cotton and maize, and there are no reports of their application in wheat for resistance to *MoT* (Daboussi et al., 2015; Zhu *et al.*, 2017; Viana *et al.*, 2019). In genetic improvement, ZFNs have been successfully used in species such as rice, maize and *Arabidopsis* (Cantos et al., 2014; Yin and Qiu, 2019; Ijaz et al., 2020). However, there is no reports of application for *MoT*.

In plant breeding, gene editing tools allow accelerating the development of new cultivars with durable resistance to pathogens by modifying loci involved in the plant’s defense system (Andolfo et al., 2016; Yin and Qiu, 2019). The applicability of these tools in wheat can be exemplified by the study in which TALEN and CRISPR/Cas9 were used to introduce simultaneous mutations in the three homologous hexaploid wheat alleles that encode proteins responsible for repressing the plant’s defenses against powdery mildew. The modification conferred broad-spectrum heritable resistance to *Blumeria graminis f. sp. tritici* (Wang et al., 2014). In another study, CRISPR / Cas9 was applied to the specific mutation in the *OsERF922* in rice, resulting in increased resistance to blast (Wang *et al.*, 2016). Despite the lack of published results on CRISPR in wheat cultivation for the development of wheat blast-resistant cultivars, it is possible to check the protocol for the application of this technology for wheat cultivation developed by Bhowmik and Islam (2020).

Gene editing tools have an important advantage over other approaches with the same purpose, as mutagenesis triggered in the host does not involve foreign DNA, which does not configure the event obtained as a transgenic. This difference can help alleviate biosafety and bioethics regulations related to genetically modified organisms, and the product tends to be better accepted by the market (Yesmin et al., 2020). Compared to mutation induction using physical and chemical agents, the advantage is that the editing tools are directed, not random. Considering all the advantages of gene editing, it is shown as a viable and sustainable alternative to develop blast resistant wheat cultivars (Yesmin *et al.*, 2020).

## Concluding remarks

Wheat is an important food grain, providing essential nutrients for humanity. The improvement has been responsible for the increase in crop yields, as well as for improving many other traits, such as grain quality, resistance to biotic and abiotic stresses. However, the aggravation of climate change requires that the improvement process be accelerated in order to keep up with the rapid advance of pathogens.

Wheat blast, a disease caused by the pathogen *Magnaporthe oryzae* pathotype *Triticum*, is considered a major threat to global cereal production due to its potential to damage the crops. Only conventional breeding efforts are not sufficient for the rapid development of new varieties with long and broad-spectrum resistance against the rapidly evolving pathogen (Figure 3).

To control this important disease, genetic resistance is considered the most efficient and sustainable way, being essential to obtain new sources of resistance. Therefore, the identification of other sources of resistance to *MoT* is of great importance. In this sense, transcription factors (TFs), which regulate the expression of genes involved in different plant functions, are strategies to identify candidate genes involved in the response to blast in wheat (Baillo et al., 2019). It is important to consider further in-depth studies to understand the transcriptional profile of target genes from TF families. Transcription factors and other candidate genes involved in blast resistance can be identified using tools such as microarrays, RNASeq and Quantitative Real-time PCR.

**Figure 4 -:**
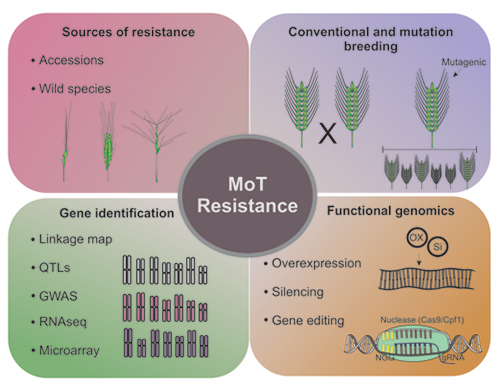
Schematic representation of plant breeding tools available to obtain wheat cultivars resistant to *Magnaporthe oryzae* pathotype *Triticum*.

Thus, besides the conventional breeding, the use of mutation breeding and technologies such as QTL mapping, gene expression analysis, transgenics and genome editing can be important approaches to assist the plant genetic improvement process (Figure 4), enabling a variety of combinations in order to slow down the co-evolution of the pathogen and improve the durability of wheat blast resistance.
